# A pilot study investigating the influence of dietary boron levels on osteoporosis in postmenopausal women

**DOI:** 10.1002/fsn3.4218

**Published:** 2024-05-19

**Authors:** Taha Rababah, Muhammad Aludatt, Sana Gammoh, Feras Bani Salameh, Ghazi Magableh, Ali Almajwal, Sevil Yücel, Yara AL‐Rayyan, Numan AL‐Rayyan

**Affiliations:** ^1^ Department of Nutrition and Food Technology Jordan University of Science and Technology Irbid Jordan; ^2^ Industrial Engineering Department Yarmouk University Irbid Jordan; ^3^ Department of Community Health Sciences, College of Applied Medical Sciences King Saud University Riyadh Saudi Arabia; ^4^ Yildiz Technical University Istanbul Turkey; ^5^ College of Agriculture and Life Sciences University of Wisconsin‐Madison Madison Wisconsin USA; ^6^ School of Medicine and Public Health University of Wisconsin‐Madison Madison Wisconsin USA; ^7^ National Agricultural Research Center Amman Jordan

**Keywords:** bone, boron, calcium, diet, osteoporosis, postmenopausal women

## Abstract

This study aimed to evaluate the impact of dietary boron on osteoporosis in postmenopausal Jordanian women. Sixty‐six women diagnosed with osteoporosis were recruited and data on personal information, dietary habits, medical history, and lifestyle were collected. Bone mineral density, serum calcium, and serum vitamin D measurements were obtained from patient records. This study showed a strong correlation between boron intake and bone mineral density in these women with osteoporosis and a negative correlation between boron intake and serum calcium (*p* < .05). However, no significant correlation was found between boron intake and various parameters such as serum vitamin D, dietary habits, body mass index (BMI), waist circumference (WC), age of menopause, cases of hysterectomy or oophorectomy, location of fractures, education level, social status, smoking, and physical activity (*p* > .05). A significant link was found between boron intake and bone mineral density highlighting the importance of nutritional and lifestyle factors affecting bone health. Further research on the specific impact of boron is warranted to better inform dietary interventions for osteoporosis prevention and management.

## Graphical abstract

This study showed a strong correlation between boron intake and bone mineral density in these women with osteoporosis and a negative correlation between boron intake and serum calcium (*p* < .05). However, no significant correlation was found between boron intake and various parameters such as serum vitamin D, dietary habits, body mass index (BMI), waist circumference (WC), age of menopause, cases of hysterectomy or oophorectomy, location of fractures, education level, social status, smoking, and physical activity (*p* > .05). The findings suggest that a national intervention program to manage osteoporosis is necessary for Jordan.

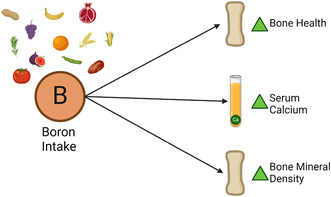

## 1 INTRODUCTION

Osteoporosis is a worldwide health concern for individuals of all ethnic and racial groups, and the incidence of osteoporotic fracture among individuals is predicted to increase with age (WHO, 2007). Annually, the number of fractures exceeds 2.3 million in the USA and Europe (WHO, 2003). In the elderly population, the overall prevalence rate of osteoporosis is 32.1%, with a higher incidence in women (55.7%) compared to men (12.4%; Porhashem et al., 2012). The worldwide lifetime risk of osteoporotic fractures ranges from 40–50% in females and 13–22% in males (Dennison et al., 2006).

Osteoporosis is typically diagnosed by measuring bone mineral density (BMD) using a central dual‐energy X‐ray absorptiometry (DEXA) scan of the total hip, femoral neck, and lumbar spine, with a T‐score of −2.5 or below indicating a diagnosis (WHO, 2003). It is a significant health issue for millions of women worldwide, with 80% of cases occurring in postmenopausal women (Abushaikha & Omran, 2010). Postmenopausal osteoporosis is the result of bone loss related to the decline in gonadal function associated with aging (WHO, 2003).

Boronic acids and derivatives, as well as other BCC, have been studied for their diagnostic and therapeutic properties, including their effects on muscle mass, basal metabolism, and fertility (Estevez‐Fregoso et al., 2023). Several studies have reported that there is an association between osteoporosis or low bone density (BMD) and alveolar bone and tooth loss (Brennan et al., 2007; Mohammad et al., 1997). Vitamin D, through both its effects on bone metabolism/BMD and the immune system, could influence the development of periodontal disease (Krall et al., 2001). Due to this potential association, vitamin D receptor gene polymorphisms have also been studied as potential genetic risk factors for periodontal disease in different ethnic populations with conflicting results so far (Park et al., 2006; Takeuchi et al., 1998). Vitamin D is mainly produced by human skin after its exposure to ultraviolet radiation through sunlight, and it is also supplied through the individual's diet (vitamins D2 and D3; Glade, 2013).

Nutrition is a modifiable pathogenic factor of osteoporosis that plays a significant role in the prevention and treatment of osteoporosis (Heaney, 1996). A patient's diet is crucial because adequate and appropriate vitamins, minerals, fats, and protein are essential for the growth and regeneration of normal tissues (Oral Health, 2019). Inadequate mineral intakes in diet, such as boron, calcium, magnesium, potassium, manganese, copper, phosphorus, fluoride, iron, zinc, and vitamins C, K, D, A, and B affect bone health, as could increase the risk of bone losses and impair bone remodeling (Naghii et al., 2006).

Calcium is one of the leading minerals that helps in bone development and helps keep a person's bones healthy and also helps to stop gum disease and tooth decay. Vitamin D is equally important to work with calcium so it can be absorbed better for stronger bones. A deficiency in this vitamin can cause what is called burning mouth syndrome (Oral Health, 2019). Vitamin D is produced in the body with sun exposure, so deficiencies are rare, but they can develop in those who do not consume milk or fish (Dragonasa et al., 2020). Vitamin D is important to work with calcium so it can be absorbed better to get healthy and stronger bones (Oral Health, 2019).

On the other hand, Boron has never been found in its elemental state in nature, conversely, it is mainly found in the form of boric acid (B(OH)_3_) or salt (borates) form (Parks & Edwards, 2005). Boric acid is used as a food preservative in certain food products, such as meats and meat products, dairy products, and caviar (Arslan et al., 2008) due to its ability to inhibit the growth of microorganisms, therefore food stays fresh longer (Normah et al., 1984). Boric acid can also be added to food products to control starch gelatinization and enhance the color, texture, and flavor. It is harmful to human health if consumed in high amounts and used in the production of food, especially noodles and some processed seafood such as fish balls (Yiu et al., 2008).

Boric acid is a water‐soluble compound and is widely distributed in body tissues, such as the brain, liver, and kidney (Murray, 1998). It is highly accumulated in the animal bone more than in blood and soft tissue (Kot, 2009; Ku et al., 1991). Therefore, boron is a vital element for bone health, and is important to maintain its precursors in adequate amounts to prevent deficiencies. Accordingly, it is considered an essential micronutrient of organisms and plays a crucial role in osteogenesis and maintenance of bone (Nielsen, 2004).

It is indicated that limited studies are conducted in Jordan to estimate the prevalence of osteoporosis (Abushaikha & Omran, 2010). Most studies focused on specific dietary components such as protein, phosphorous, calcium, and vitamins (Masse et al., 2004). Although calcium and vitamin D have a direct effect on bone health, many other nutrients and food constituents can affect bone health, thus a balanced diet is necessary for bone health and any modification is recommended to reduce the osteoporosis risk (Gueldner et al., 2008).

Consequently, the purpose of this study was to assess the effect of dietary boron on osteoporotic postmenopausal women in Jordan. In addition to calculating the daily intake of boron and calcium by the participants, through investigation of boron content in some foods and herbs consumed frequently by them.

## 2 MATERIALS AND METHODS

### 2.1 Subject selection

A total of 66 participants of Jordanian postmenopausal women were chosen on a voluntary basis from Royal Medical Services Hospitals between February and September 2018. The age of women was 41–77 years as premenopausal women are more susceptible to developing osteoporosis due to a lack of gonad hormones. The participants were previously diagnosed with osteoporosis and BMD was 2.5 SD below the mean of young healthy adults as determined by dual‐energy X‐ray absorptiometry (DEXA) scans.

### 2.2 Study survey

We adapted a questionnaire (see Appendix A) from a survey by the Jordanian Osteoporosis Prevention Society (JOPS) to meet the objectives of our study. The questionnaire aimed to explore the effect of dietary boron on osteoporotic postmenopausal women by gathering demographic data, such as age, educational level, monthly income, marital status, and medical history (including diseases, medication, and supplements), as well as boron intake and consumption among participants with different lifestyle behaviors (such as physical activity, cigarette smoking, daily meals and snacks, calcium intake, and specific types of fruits and vegetables, herbs, and seeds).

Participants were interviewed and the purpose of the study was explained to obtain their consent. Data were collected and recorded during the distribution of the questionnaires in the interviews.

### 2.3 Body mass index (BMI)

Weight was measured and recorded in kilograms using an advanced Tanita scale MC‐780U, while height was measured and recorded in meters from the top of the head to the sole of the feet. BMI was calculated as weight in kilograms divided by height in meters squared (kg/m^2^).

Waist circumference was measured with a measuring tape without compressing the skin. Participants were instructed to stand straight with their feet together and their abdomen in a relaxed state.

### 2.4 Food samples

Fruits and vegetables were purchased from the local market, and seeds were purchased from the spice market between June and August for analysis (see Table 1).

**TABLE 1:** Food samples.

Food category	Number	Food item
Fruits	Nine	Grapes, orange, peach, plums, banana, dates, apricot, cherry, and figs
Vegetables	Six	Tomato, potato, spinach, cabbage, red cabbage, and lemon
Seeds	Eight	Soybean, corn, sesame, broad bean, wheat, coffee, peanut, and nigella sativa

### 2.5 Preparation of food samples

The food samples were prepared according to the method reported by Webb et al. (2002) before analysis. A composite sample of 1 kg of each food item was homogenized by either crunching or mincing. Then, 1.0 g of each item was weighed into a beaker that had been rinsed with acid. Next, 50 mL of demineralized water (distilled) was added, and the mixture was boiled for 10 min. The contents of the beaker were filtered using filter paper (Whatman, 125 mm) into a 100‐mL volumetric flask. To stabilize the solution, 1 mL of concentrated nitric acid was added, and the volume was made up to the 100 mL mark with distilled water. The solution was then stored in a polyethylene flask until further analysis.

All chemicals used were of analytical reagent grade and were obtained from Sigma Chemical Company and Merck, Darmstadt, West Germany. The standard was brought from Polyscience Inc., Niles, Illinois, USA.

### 2.6 Analytical method

Boron concentration in the food samples was determined using the hot water extraction method described by Webb et al. (2002). Boron concentration in the samples was determined according to the method described by the American Water Works Association (AWWA, 2006). A standard serial dilution of pure boron number S6615433 (Merck company) was used to prepare the calibration curve. To prepare the working standard, 1 mL of the standard stock (1000 ppm) was dissolved in a 100‐mL volumetric flask and diluted to volume with 100‐mL demineralized water to prepare a 10 ppm working standard. The working standard was used to prepare standards of 0.05, 0.1, 0.2, 0.4, 0.8, and 1.0 ppm by diluting 0.5, 1, 2, 4, 8, and 10 mL of the working standard in 100‐mL volumetric flasks with distilled water.

The reference standard (0.3 ppm) was prepared to be used in this assay. The digested boron of the prepared samples of each item was quantitatively measured using an inductively coupled plasma mass spectrometer (ICPM 8500; Shimadzu, Kyoto, Japan), applying the instructions described by Faires et al. (1984). The ICPM was operated with a flowing stream of argon gas ionized by an applied radio frequency field oscillating at 27.1 MHz, at a temperature of 8000°K, and a wavelength of 249.77 nm.

### 2.7 Assessment of dietary boron intake

Daily dietary boron intake was determined according to the methods described by Rainey et al. (1999) with some modifications. The quantity of boron‐containing foods consumed by each participant, including fruits, vegetables, seeds, and herbs, was multiplied by the boron concentration of each item to calculate the daily dietary boron intake.

### 2.8 Assessment of dietary calcium intake

Estimation of daily dietary calcium intake was conducted using a modified method based on a previous study by Pritchard et al. (2010). The method involved multiplying the amount of calcium‐rich foods consumed by the participant, such as milk, yogurt, cheese, and sardines, with their corresponding calcium concentrations of 275, 275, 200, and 380 mg, respectively (USDA, 2011). In addition, as the targeted group took 500 mg of calcium supplements daily, this amount was added to their total calcium intake.

### 2.9 Statistical analysis

Statistical analysis was conducted using the Statistical Package for Social Sciences software (SPSS, version 19, Chicago Inc). Descriptive statistics were used to summarize the characteristics of the study population, including frequency distribution for categorical variables and mean and standard deviation for continuous variables. A *p*‐value of ≤.05 was considered statistically significant.

Dietary intakes of boron were grouped and the distribution of categorical variables across boron intake categories was tested using the chi‐square test. One‐way analysis of variance (ANOVA) was used to investigate the impact of variables of interest, and the least significant difference (LSD) posthoc ANOVA was conducted to determine the differences between variable groups. A *p*‐value of ≤.05 was considered statistically significant for both ANOVA and LSD posthoc ANOVA. The LSD posthoc ANOVA was also used to determine the differences between boron concentrations.

## 3 RESULTS AND DISCUSSION

### 3.1 General participant's characteristics

The general characteristics of 66 postmenopausal women with osteoporosis who participated in this study are presented in Table 2. The mean age of participants was 59.1 ± 9.1 years, with an age range between 41 and 77 years. Our results showed that the mean menopausal age was 47.8 ± 4.6 years, which is relatively higher than the mean age of menopause at 46.7 years of some other studies (Hidayet et al., 1999). A previous study conducted on postmenopausal women with osteoporosis reported an average age between 48 and 59 years old (Kröger et al., 1994).

**TABLE 2:** General characteristics of participants where the results presented as a mean value or (%).

Variable	*n*	% mean ± SD
Age (years)	66	59.13 ± 9.13
Age at menopause	66	47.8 ± 4.61
Height (cm)	66	157.42 ± 0.06
Weight (kg)	66	73.09 ± 15.20
Categories of BMI (kg/m^2^)^a^	66	
Underweight	0	0
Normal weight	14	21.21
Overweight	26	39.39
Obese	24	36.36
Morbid obesity	3	4.54
Waist circumference (cm)^b^	66	92.31 ± 16.28
Normal	29	43.94
Abnormal	37	56.06
BMD^c^	66	−2.99 ± 0.24
Daily boron intake (mg)	66	3.3229 ± 0.6489
Daily boron intake (mg) from		
Fruits and vegetables	66	55.38
Seeds	66	34.65
Herbs	66	9.69
Calcium blood level (mg/dL)^d^	66	7.48 ± 1.12
Vitamin D (nmol/L)^e^	66	14.32 ± 5.08
Educational level		
Illiterate	13	19.69
Primary school	14	21.21
Secondary school	20	30.30
Diploma	13	19.69
Bachelor	6	9.09
Marital status		
Married	41	62.12
Single	1	1.51
Divorce	5	7.57
Widow	19	28.79
Family income		
>200 JD	19	28.79
200–500 JD	28	42.42
<500 JD	19	28.79
Physical activity		
Low	47	71.21
Moderate	19	28.79
Smoking cigarettes		
Current smoker	8	12.12
Past smoker	3	4.54
Nonsmoker	55	83.33
Snacks daily intake		
1 meal	15	22.72
2 meals	30	45.45
3 meals	14	21.21
<3 meals	7	10.60
Breakfast intake		
Daily	43	65.15
3–4 times/week	16	24.24
Rarely	7	10.60
Shortage in current length		
Yes	53	80.30
No	13	19.70
Frequently fall		
Yes	20	30.30
No	46	69.70
Age when fracture		
>30	3	4.54
30–40	14	21.21
41–50	26	39.39
<50	17	25.75

*Note*: Data are presented as mean ± standard deviation (SD) or as percentages.

^a^ Body mass index (BMI): underweight (BMI < 18.5 kg/m^2^); normal weight (BMI between 18.5 and 24.9 kg/m^2^); overweight (BMI between 25 and 29.9 kg/m^2^); obese (BMI between 30 and 39.9 kg/m^2^); morbid obesity (BMI 40 and above).

^b^ WC values (normal: <88 cm, abnormal: ≥88).

^c^ Bone mineral density BMD (normal: T‐score >−1, osteopenia: T‐score −1 to −2.5, osteoporosis: T‐score <−2.5).

^d^ Normal calcium level 8.5–10.5 mg/dL.

^e^ Vitamin D level (sever deficiency: <12.5 nmol/L, moderate deficiency: ≥12.5 to <25 nmol/L, mild deficiency: ≥25 to <50 nmol/L, insufficiency: ≥50 to ≤75 nmol/L, adequate: >75 nmol/L, desirable: >100 nmol/L).

Our results indicated that the majority of participants (71.2%) had low physical activity. This agrees with a study that reported that exercise is considered an important preventive factor for osteoporosis, leading to more bone remodeling and increased bone gain (OBrien, 2001). Therefore, structured exercise programs for osteoporotic individuals are required and must be carefully planned in collaboration with a physician (Dalsky et al., 1988). However, a study revealed that moderate to vigorous physical activity is associated with a hip fracture risk reduction of 45% (Moayyeri, 2008).

The majority (83.3%) of the participants were nonsmokers. Some other studies consider smoking as a risk factor for developing osteoporosis, but our study did not show an association between smoking and osteoporosis (Kanis et al., 2005).

Regarding marital status, 62.1% of participants were married, and 42% had a family income between 200 and 500 JD. Another study showed the same relationship between low income and the incidence of osteoporosis (Batieha et al., 2011).

All participants consumed boron dietary sources with a mean daily intake of 3.32 ± 0.65 mg from natural dietary sources. The food categories with the highest boron content were 55.38% fruits and vegetables, 34.65% seeds, and 9.96% herbs. Consequently, the boron intake of participants fit within the range of the European boron daily intake which ranges from 1.7 to 7.0 mg per day (Richold, 1998). However, the range was higher than that reported in Australia (2.2–2.3 mg per day; Rainey & Nyquist, 1998).

Moreover, the mean daily calcium intake among participants was 1059 ± 202 mg, which is lower than the recommended dietary allowance (RDA) of calcium for females aged 51–70 years (1200 mg), and higher than the RDA for the ages 19–50 years (1000 mg; CRDRIVDC, 2010).

In addition, the results in Table 2 showed that 22.7, 45.5, 21.2, and 10.6% of participants reported consuming snacks 1, 2, 3, and >3 times daily, respectively. Meanwhile, 65.2% reported consuming breakfast daily, 24.3% reported eating breakfast 3–4 times per week, and 10.6% rarely consumed breakfast. The majority of participants (56%) reported consuming three meals per day, followed by 39.4% consuming two meals per day, 3% consuming four meals per day, and 1.5% consuming only one meal per day. These findings agree with a study emphasizing the importance of consuming three meals or more per day for health (McCrory & Campbell, 2011).

The results revealed that fracture incidence varied across different age groups, with 39.4, 25.8, 21.2, and 4.5% of participants experiencing fractures within the ages of 41–50, <50, 30–40, and >30 years, respectively. These findings differ from a previous study that reported that women between the ages of 75–95 years were 20 times more susceptible to fractures than those aged 50–54 years old (Maalouf et al., 2013). It is worth noting that the incidence of fractures is influenced by a range of factors, including age, gender, and region (Gullberg et al., 1997). Additionally, the majority of participants (69.7%) did not experience frequent falls, while only 30.3% were susceptible to frequent falls.

### 3.2 Effect of dietary habits on boron intake

The results of Table 3 revealed no significant (*p* > .05) correlation between the number of meals consumed by participants and boron intake. Similarly, the relationship between breakfast meal intake and boron intake was also insignificant (*p* > .05). It is worth noting that while there was no correlation between breakfast intake and boron intake, nutritionists emphasize breakfast for human health.

**TABLE 3:** Participant's dietary habit effect on boron intake (mg) among participants.

Variable	Boron intake (mg)	*p*‐value
Number of meals	1	3.598 ± 0
	2	3.391 ± 0.714
	3	3.271 ± 0.638
	4	3.252 ± 0.180
Breakfast intake	Daily	3.33 ± 0.662
	3–4 times/week	3.289 ± 0.656
	Rarely	3.356 ± 0.694
Number of snacks	1	3.560 ± 0.631
	2	3.124 ± 0.656
	3	3.468 ± 0.630
	4	3.43 ± 0.590
Age		
40–50	3.560 ± 0.631	.251
51–60	3.124 ± 0.656	
61–70	3.468 ± 0.630	
70<	3.434 ± 0.591	
Education levels		
Illiterate	3.350 ± 0.732	.376
Primary school	3.623 ± 0.678	
Secondary	3.228 ± 0.605	
Diploma	3.187 ± 0.671	
Bachelor	3.170 ± 0.484	
Family income (JD)		
Less than 200	3.226 ± 0.502	.566
200–500	3.422 ± 0.781	
More than 500	3.272 ± 0.589	
Social status		
Married	3.353 ± 0.698	.106
Single	3.237 ± 0.0	
Divorce	3.902 ± 0.424	
Widow	3.108 ± 0.526	
Smoking		
Yes, currently smoking	3.302 ± 0.735	.958
Yes, In past	3.429 ± 0.857	
No, I did not smoke at all	3.320 ± 0.645	
Physical activity		
Low	3.229 ± 0.653	.67
Moderate	3.554 ± 0.610	

*Note*: All values are mean ± SD. Values determined with ANOVA (*p* < .05).

Regarding the number of snacks consumed, participants reported consuming 1, 2, 3, or 4 snacks per day. However, the mean value of boron intake across the different snack frequencies was found to be insignificant (*p* > .05), as shown in Table 3.

Calcium fructoborate (CaFB), the most extensively studied fructoborate, is a naturally occurring sugar–borate complex found in commonly consumed herbs, vegetables, fruits, seeds, and nuts (Hunter et al., 2019). It has been clinically demonstrated to significantly reduce joint discomfort and improve flexibility (Hunter et al., 2019). It is noteworthy that there were no studies found in the literature that specifically addressed the relationship between snack consumption and boron intake, making our study a novel contribution to this field.

Our findings suggest that dieticians should consider incorporating healthy snack options into participants' diets, especially as the majority of participants reported enjoying snacks. This may not only increase the likelihood of meeting adequate boron intake levels but also contribute to overall dietary health. It is important to note, however, that further research is needed to confirm these results and to better understand the relationship between dietary habits and boron intake.

### 3.3 Association between menopausal age and BMD

**FIGURE 1:**
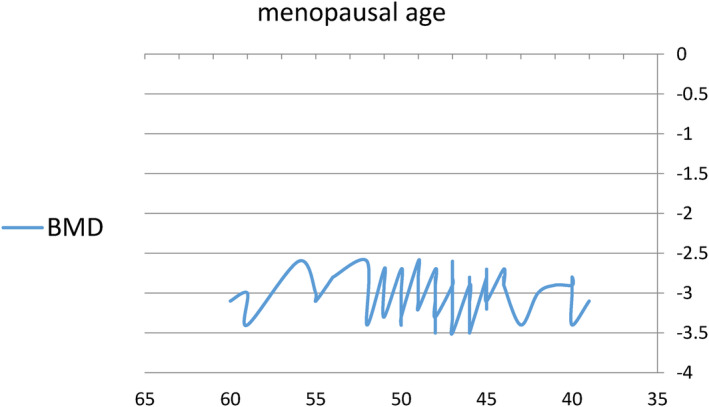
The association between menopausal age and bone mineral density (BMD) expressed as T‐scores as measured by dual‐energy X‐ray absorptiometry (DEXA) of participants.

The menopausal age of participants ranged from 39 to 60 years, with a mean value of 47.8 ± 4.6 years. The T‐score for the BMD of participants ranged from −2.6 to −3.5 (Figure 1). Our results showed no significant correlation between menopausal age and BMD. This may be due to the complex interplay of multiple nutrients that affect BMD (Gueldner et al., 2008), which were not taken into account in this study. Further research is needed to fully understand the factors that influence BMD in postmenopausal women.

### 3.4 Effect of boron daily intake on BMI, WC, and BMD

Table 4 presents the mean values of boron intake, BMI, waist circumference, and BMD among three groups that have different amounts of boron intake. The results indicate no significant correlation (*p* > .05) between boron intake (mg) and BMI or waist circumference.

**TABLE 4:** The effect of boron intake (mg) on BMI and WC of Jordanian osteoporotic postmenopausal women aged 41–77 years.

Participants	Mean boron intake (mg)	BMI	WC	BMD
Group 1	≤2.979	28.76 ± 4.9	90.50 ± 15.4	−3.205 ± 0.20^a^
Group 2	2.980–3.573	29.05 ± 6.0	91.36 ± 17.8	−2.995 ± 0.21^b^
Group 3	≥ 3.574	30.22 ± 5.1	95.09 ± 16.3	−2.795 ± 0.13^c^
*p*‐value		.517	.627	.000

*Note*: All values are mean ± SD. Values determined with ANOVA (*p* < .05) after adjustment for age, drugs, BMI, related diseases, surgery, menopause age, and calcium intake.

Values with different letters are significantly different (*p* < .05)

However, a strong significant (*p* < .05) relation is observed between boron intake and BMD. This finding is consistent with a previous study that has reported on the beneficial effects of boron on cortical bone strength and trabecular bone micro‐architecture (Nielsen & Stoecker, 2009). Additionally, a study conducted on rabbits reported that boron has positive effects on bone strength and mineral composition (Hakki et al., 2013). This suggests that boron supplements could be useful for the treatment of osteoporosis and in maintaining the bone health of women in the future (Scorei, 2013).

The positive relation between daily boron intake and BMD is further supported by the amount of daily boron intake, which ranged from 2.3 to 4.8 mg, with a mean value of 3.3 ± 0.65 mg. The T‐score for BMD of participants ranged from −2.6 to −3.5, indicating the potential importance of boron intake for maintaining bone health. The positive effects of boron on BMD could be due to its ability to modify mineral amounts in bones or regulate certain hormones involved in bone growth (Basoglu et al., 2010; Palacios, 2006).

**FIGURE 2:**
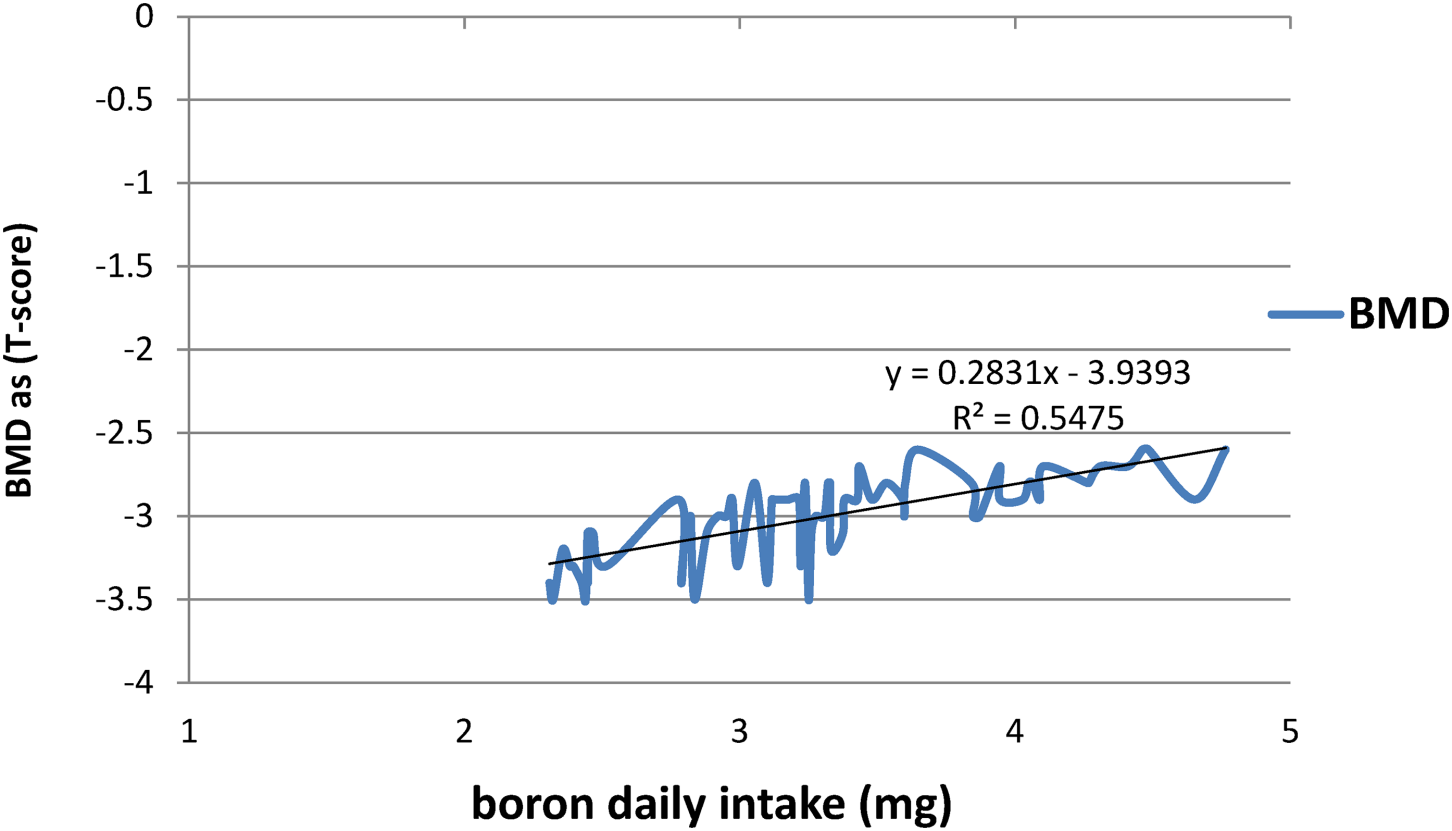
The relation between boron daily intake (mg) and bone mineral density (BMD) expressed as T‐scores as measured by DEXA among participants.

Overall, the results suggest that higher boron intake could have beneficial effects on BMD (Figure 2), and further research is warranted to explore the potential role of boron supplements in promoting bone health.

### 3.5 Boron concentration

Table 5 displays the boron concentrations (ppm) in various food items including fruits, vegetables, cereals, and herbs. The highest concentrations of boron were found in figs, thyme, grapes, dates, plums, soybean, and mint with concentrations of 15.8, 14.9, 12.8, 10.9, 10.3, 9.9, and 8.3 ppm, respectively. Figs had the highest mean value of 15.75 ppm among the tested items. Additionally, the mean value of plums (10.3 ppm) in this study was found to be higher than the mean values of Australian (4.5 ppm), Turkish (Naghii et al., 1996) (1.16 ppm) (Sungur & Okur, 2009), American (4.22 ppm) (Anderson et al., 1994), Finnish (4.5 ppm) (Koivistoinen, 1980), and German (3.4 ppm) plums (Souci et al., 1994). The lowest concentration of boron was found in coffee (0.92 ppm), which was lower than the 3.28 ppm reported in a previous study for coffee (Krejcova & Cernohorsky, 2003).

**TABLE 5:** Average boron concentration (ppm) in various food items collected from different local markets in Jordan.

Food item	Bone concentration (ppm)
	Mean ± SD
Figs	15.75 ± 0.919a
Thyme	14.9 ± 0.990b
Grapes	12.8 ± 0.849c
Dates	10.9 ± 0.566d
Plums	10.315 ± 0.686de
Soybean	9.89 ± 0.580e
Mint	8.32 ± 0.424f
Parsley	5.82 ± 0.269g
Nigella sativa	5.4 ± 0.283g
Peanut	5.93 ± 0.184g
Corn	5.675 ± 0.177g
Peach	4.605 ± 0.163h
Horsetail	4.38 ± 0.283h
Wheat	3.84 ± 0.085hi
Spinach	3.595 ± 0.120ij
Banana	3.26 ± 0.919ijk
Dandelion	3.265 ± 0.990ijk
Orange	2.965 ± 0.849jkl
Rocket	3.01 ± 0.566jkl
Potato	2.665 ± 0.686klm
Tomato	2.505 ± 0.580klmn
Green tea	2.49 ± 0.424klmno
Pomegranate	2.255 ± 0.269lmno
Sesame	2.355 ± 0.283a
Red cabbage	1.96 ± 0.184b
Broad bean	1.795 ± 0.177c
Apricot	1.71 ± 0.163d
Cabbage	1.335 ± 0.283de
Lemon	1.185 ± 0.078e
Coffee	0.92 ± 0.057f

*Note*: Mean ± SD, values with the same letters are not significantly different (*p* ≤ .05).

### 3.6 Association of boron intake and clinical characteristics

Table 6 displays the location and percentage of fractures among three groups of participants. The total percentage of fractures for all groups was 13.6%, 28.7%, 1.5%, 30.3%, and 16.6% in the cases of vertebral, hip, ribs, wrist, and multifracture, respectively. Moreover, 9% of participants did not experience any fractures, whereas 91% of participants experienced at least one fracture, with the dominant location being at the wrist and hip. These results differ from a previous study that showed that wrist fracture was 32% and vertebral fracture was 60% among osteoporosis cases (Sellami et al., 2006). Additionally, only 13.6% of participants developed vertebral fractures, which may be related to their consumption of an adequate amount of boron food sources. This finding is consistent with a previous report that indicates that boron increases vertebral resistance to crushing force (Chapin et al., 1998). However, although the results showed lower fracture cases, there was no significant correlation (*p* > .05) between daily boron intake and fracture location.

**TABLE 6:** Association of boron intake (mg) and some clinical characteristics of osteoporotic Jordanian postmenopausal women aged 41–77 years.

Variables	Group 1	Group 2	Group 3	*p*‐value
Fracture location				
No fracture	1 (1.5%)	2 (3%)	3 (4.5%)	.448
Vertebra	2 (3%)	2 (3%)	5 (7.6%)	
Hip	9 (13.6%)	7 (10.6)	3 (4.5%)	
Rips	0 (0.0%)	0 (0.0%)	1 (1.5%)	
Wrist	5 (7.6%)	7 (10.6%)	8 (12.1%)	
<One location	5 (7.6%)	4 (6%)	2 (3%)	
Hysterectomy				
Yes	2 (3%)	4 (6%)	2 (3%)	.566
No	20 (30.3%)	18 (27.8%)	20 (30.3%)	
Oophorectomy				
Yes	6 (9%)	3 (4.5%)	4 (6%)	.511
No	16 (24.2%)	19 (28.8%)	18 (27.8%)	
Age of menopause				
>45	5 (7.6%)	8 (12.1%)	9 (13.6%)	.730
46–55	15 (22.7%)	13 (19.7%)	12 (18.2%)	
<55	2 (3%)	1 (1.5%)	1 (1.5%)	

*Note*: Data are presented as *n* (%), number of participants (percentages). Values determined with ANOVA (*p* < .05).

In addition to fracture location, the study also recorded boron intake among participants and the percentage of those who underwent hysterectomy and oophorectomy operations. Results showed that most participants (88%) did not undergo a hysterectomy operation or oophorectomy (80%). Previous studies on the effect of applying poly‐(lactide‐co‐glycolide) scaffolds containing boron nitride and hydroxyapatite on bone defects in osteopathic rates found that improved healing occurred in some concentrations, suggesting that focused targeting therapies may be beneficial for enhancing bone regeneration and repair (Topcu et al., 2024).

Furthermore, the mean calcium daily intake among participants was 1059 ± 202 mg, which is lower than the mean value of the recommended daily allowance (RDA) of calcium for females at the age of 51–70 years (1200 mg) and higher than the RDA for the age of 19–50 years (1000 mg; CRDRIVDC, 2010). The results showed that the majority (60.6%) of menopausal ages were within the age range of 46–55 years, and very few participants (6%) reported a menopausal age <55 years. However, there was no significant (*p* > .05) association between boron intake and the age of menopause.

### 3.7 Daily calcium intake

**FIGURE 3:**
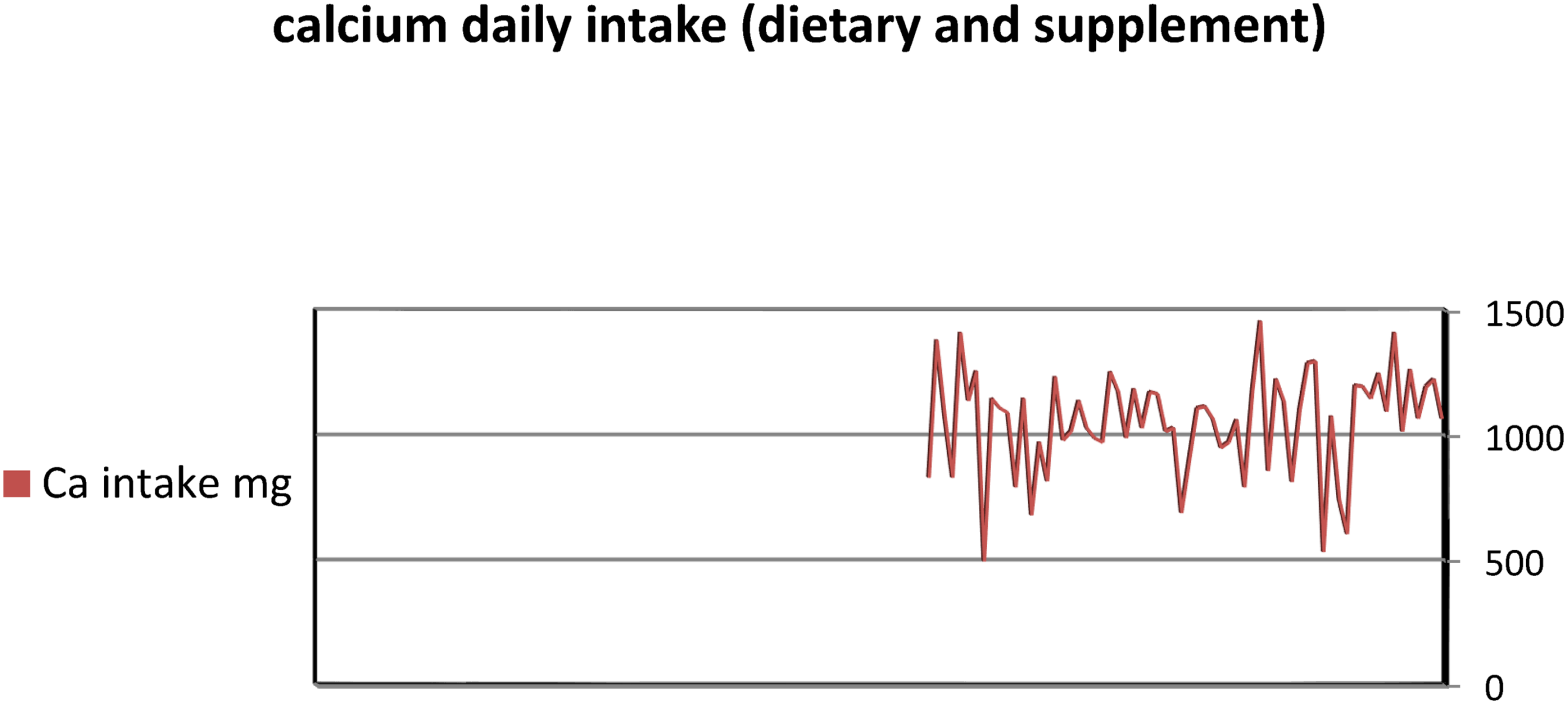
The amount of calcium daily intake of both dietary and supplements.

The mean value of daily calcium intake among participants was 1059 ± 202 mg per day, ranging from 500 mg to 1459 mg per day (Figure 3). This mean value represents the combination of dietary calcium and calcium supplement intake, with a dose of 500 mg per day. Thus, there was a wide range of daily calcium intake among participants. However, this mean value is lower than the recommended calcium intake of 1200 mg per day for individuals in the US who are over 50 years old and higher than the recommended daily calcium intake of 800 mg for individuals in Scandinavia (Nordic Council of Ministers, 2004; Yates et al., 1998).

Given the mean value of dietary calcium intake of 558 ± 202 mg per day, it is considered insufficient. Since most participants obtain their calcium from dietary sources, it is essential to introduce calcium supplements to their daily diet to maintain bone health. Otherwise, it will be challenging to prevent osteoporosis. Nevertheless, our findings suggest high calcium consumption, which agrees with a previous study that found a higher occurrence of osteoporosis and hip fractures in countries with high calcium intake (Abelow et al., 1992).

### 3.8 Relation between daily calcium intake and BMD

**FIGURE 4:**
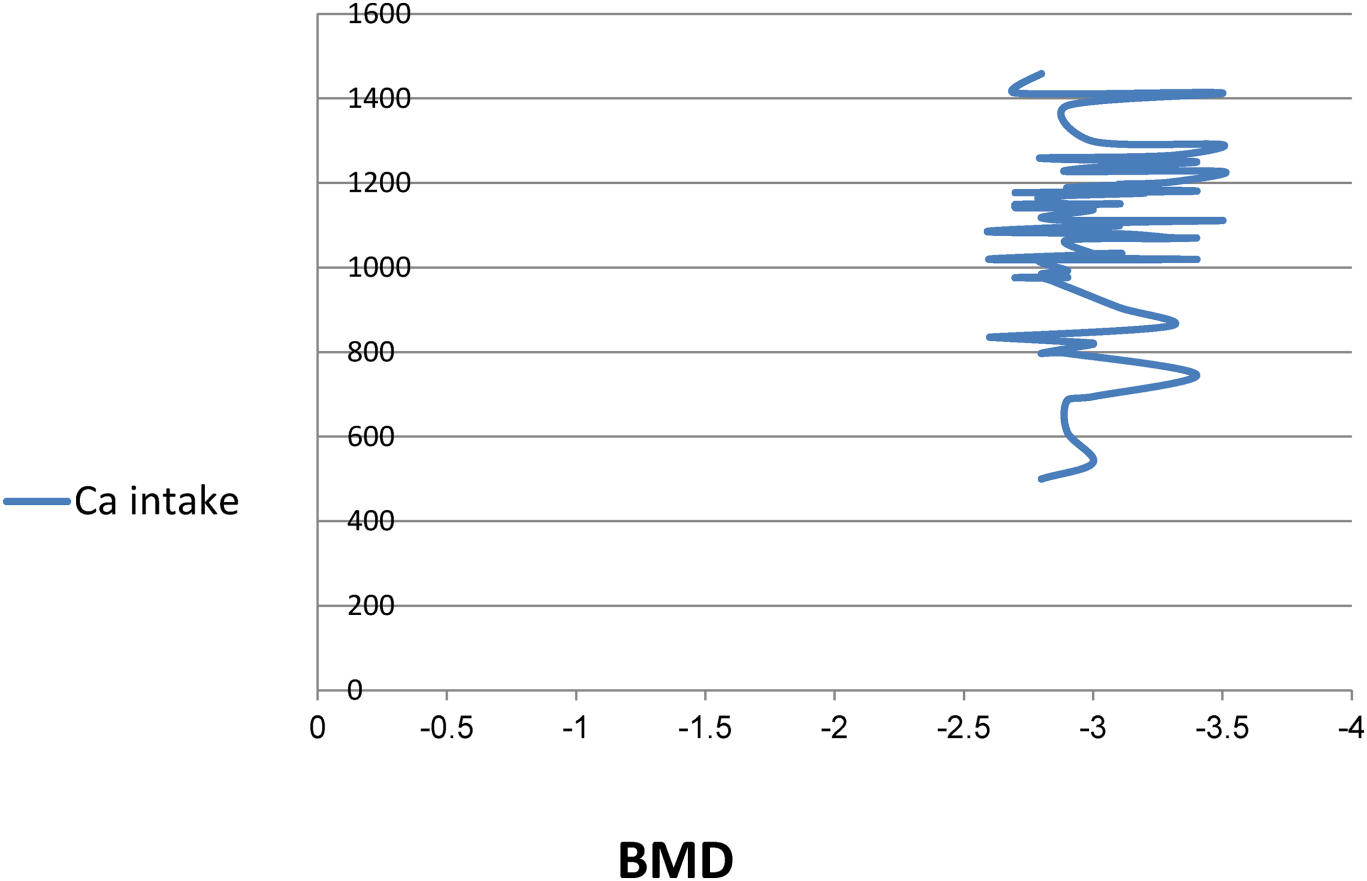
The relation between calcium daily intake (mg) and BMD.

The results showed no association between daily calcium intake and BMD (Figure 4). This finding is consistent with a previous study (Angus et al., 1988). However, a positive correlation between dietary calcium intake and BMD was observed in postmenopausal women (Kanders et al., 1988).

The poor association observed in this study could be attributed to several factors. Firstly, estimating calcium intake for a long period can be challenging, leading to misreporting by some participants. Additionally, some participants may have skipped taking their calcium supplements, potentially leading to a reduced calcium intake. Finally, other nutrients besides calcium can also impact BMD, which could have influenced the results.

### 3.9 Effect of boron intake on serum calcium and serum vitamin D

The effect of boron intake on serum calcium and serum vitamin D levels among three groups of participants is presented in Table 7. The results reveal that boron intake has a significant negative correlation with serum calcium levels (*p* ≤ .05). However, the effect of boron on urinary calcium balance as indicated in many studies revealed conflicting data. Others reported that there was no effect of boron on urinary calcium excretion in postmenopausal women when dietary boron intake was increased by 3 mg/dL (Beattie & Peace, 1993). Moreover, others showed that there is no association between urinary boron and calcium excretion (Sutherland et al., 1999).

**TABLE 7:** The effect of boron intake (mg) on serum level of calcium and vitamin D of participants.

Participants	Boron intake (mg)	Serum calcium (mg/dL)	Serum vitamin D (nmol/L)
Group 1	≤2.979	8.03 ± 0.84^a^	15.45 ± 4.9
Group 2	2.980–3.573	7.38 ± 1.2^b^	14.43 ± 5.6
Group 3	≥3.574	7.03 ± 1.1^b^	13.09 ± 4.6
*p*‐value		.009	.306

*Note*: All values are mean ± SD. Values determined with ANOVA (*p* < .05).

Values with different letters are significantly different (*p* < .05)

On the other hand, others observed a decline in urinary calcium excretion in men with boron supplements (Naghii & Samman, 1997). In addition to that, boron may interact with steroid hormones, and be involved in the prevention of calcium loss and bone demineralization, it has been shown that boron supplementation markedly reduces urinary calcium excretion and increases serum levels of estradiol hormones (Nielsen et al., 1987). Moreover, it increases calcium absorption in postmenopausal women (Beattie & Peace, 1993).

It should be noted that although there is no relationship between boron intake and serum calcium, serum calcium cannot be considered an indicator of whole‐body calcium status, as the body can maintain serum calcium homeostasis through various mechanisms, including an increase in intestinal absorption, a decrease in urinary excretion, and an increase in bone resorption (Lee & Nieman, 2009; Mahan et al., 2012).

There was no significant correlation between boron intake and serum vitamin D levels (*p* > .05), which contradicts a previous study that reported boron to function as a growth stimulator for vitamin D‐deficient animals and alleviate perturbations in mineral metabolism that are characteristic of vitamin D deficiency (Hunt, 1994).

In conclusion, diet modification is recommended to reduce the risk of osteoporosis, and a balanced diet is necessary for bone health and development. It is important to note that not only calcium and vitamin D but also many other nutrients and food constituents can affect bone health (Sungur & Okur, 2009).

## 4 CONCLUSION

In conclusion, our study highlights the potential of boron, naturally occurring in various foods, as a preventive element against osteoporosis in postmenopausal women by enhancing bone mineral density (BMD). While we identified foods with high boron concentrations, no significant correlation was found between boron intake and several parameters, including dietary habits, serum vitamin D levels, BMI, and fracture locations. Additionally, daily calcium intake showed no correlation with BMD. Notably, a strong correlation was observed between boron intake and BMI among osteoporotic postmenopausal women. Risk factors such as low physical activity, high BMI, elevated waist circumference, low serum vitamin D levels, and smoking were also noted. The findings underscore the importance of early‐life preventive measures to optimize bone health and the role of healthcare providers and nutritionists in guiding dietary and lifestyle modifications for at‐risk populations.

## AUTHOR CONTRIBUTIONS

**Taha Rababah:** Conceptualization (equal); investigation (supporting); writing – original draft (equal). **Muhammad Aludatt:** Investigation (supporting); methodology (equal). **Sana Gammoh:** Formal analysis (equal); validation (equal). **Feras Bani Salameh:** Investigation (equal); resources (equal); writing – original draft (supporting). **Ghazi Magableh:** Data curation (supporting); resources (equal). **Ali Almajwal:** Formal analysis (supporting); funding acquisition (equal). **Sevil Yücel:** Methodology (equal). **Yara AL‐Rayyan:** Data curation (equal); writing – review and editing (equal). **Numan AL‐Rayyan:** Data curation (equal); resources (equal); writing – review and editing (equal).

## FUNDING INFORMATION

The support provided by the Deanship of Research (210‐2014) at Jordan University of Science and Technology is appreciated. The authors extend their appreciation to the Researchers Supporting Project number (RSP2024R502), King Saud University, Riyadh, Saudi Arabia for funding this project.

## CONFLICT OF INTEREST STATEMENT

The authors declare that they have no conflicts of interest related to the data presented in this article.

## Data Availability

Data are available online at the following link: https://data.mendeley.com/datasets/kr438rxt4s/1.

## APPENDIX A QUESTIONNAIRE

### A.1

**Jordan University of Science and Technology**

**Faculty of Agriculture**

**Nutrition and Food Technology Department**

**Questionnaire**

Note: this questionnaire aims to evaluate osteoporosis, the risk of fracture, and other factors that may contribute to them and also to study the effect of boron content in food and herbs on osteoporotic postmenopausal women. All information will be treated in full secret.

**First part:** Personnel information section

1. Name:

2. Age:

3. Telephone number:

4. Educational level:

(1) illiterate (2) primary school (3) secondary school (4) diploma (5) bachelor (6) PHD

5. Marital status:

(1) married (2) single (3) divorced (4) widow

6. Monthly family income:

(1) Less than 200 JD (2) 200–500 JD (3) more than 500 JD

7. Age of menopause ………….

8. Do you note any shortage in your current height than yours of 20 or 30.

(1) yes (2) no

**Second part:** Medical history section

1. Do you have any of the following disease:

**Table:**

Number	Disease	Yes	No
1	Type 1 diabetes		
2	Type 2 diabetes		
3	Hypertension		
4	Hyperlipidemia		
5	Heart disease		
6	Hyperthyroidism		
7	Hyperparathyroidism		
8	High cortisone secretion		
9	Chronic rheumatism		
10	Bronchitis		
11	Renal failure		
12	Hypothyroidism		
13	Peptic ulcer		
14	Other disease mention……………………………………………		

2. Have you taken any of the following drugs for 3 months or more:

**Table:**

Number	Drugs	Yes	No
1	Thiazides (diuretic)		
2	Heparin		
3	Birth control pills		
4	Thyroid hormone pills		
5	Anti‐vit. K (anticlotting)		
6	Corticosteroid (5 mg or more daily cortisone)		
7	Antihyperglycemia		
8	Antihypertension		
9	Antihyperlipidemia		
10	Analgesic "Painkiller"		
11	Antiacid		
12	Weight Reducing		
13	Osteogrow, Alendamox		
14	Other drugs		
15	I don't take any drug		

3. Have any of your parents experience fracture?

(1) Vertebra (2) hip (3) rips (4) wrist (5) other, mention ……………

4. Have any of your brothers experience fracture?

(1) Vertebra (2) hip (3) rips (4) wrist (5) others, mention ……………

5. Have you experienced fracture?

(1) Vertebra (2) hip (3) rips (4) wrist (5) multiple fractures (0) no fracture

6. Do you frequently fall? (1) yes (2) no

7. At what age did your fracture occur?

(1) Less than 30 (2) 30–40 (3) 40–50 (4) more than 50

8. Have you had a hysterectomy before 45 years? (1) yes (2) no

9. Have you undergone ovariectomy (Oophorectomy) before 45 years? (1) yes (2) no

10. Do you take calcium supplements? (1) yes (2) no

11. Do you take vitamin D supplements? (1) yes (2) no

12. Your calcium blood test result…………

13. Your vitamin D blood test result…………

14. Your (DEXA) results …………………

**Third part**: Lifestyle behavior section

1. Your physical activity? (1) low (2) moderate (3) high

2. Have you ever smoked a cigarette or Argeelah

3. Current smoker, on average how many cigarettes daily ………….Ex‐smoker nonsmoker How long have you exposed to other smoking (ex‐smoking)

(1) Less than 3 h (2) 3–8 h (3) 9 h (4) not exposed to smoking

4. Have you ever drunk alcohol? (1) yes, how much………… (2) no

**Fourth part:** Dietary habit section

1. Height
2. Weight
3. Body mass index (BMI)
4. Waist circumference
5. Do you note any weight changes? (1) yes (2) no
  If your answer is yes, the change is … kg, (1) increment (2) weight reduction
6. How many meals do you consume daily?
  (1) 1 meal (2) 2 meals (3) 3 meals (4) more than 3 meals
7. Do you take breakfast?
  (1) Daily (2) 3–4 times per week (3) rarely
8. How many times do you consume snacks daily?

(1) 1 meal (2) 2 meals (3) 3 meals (4) more than 3 meals

9 What type of snacks you may consume (can choose many answers)
  (1) Chips (2) chocolate (3) dessert (4) fresh juices (5) salty products (6) fruits (7) ice cream (8) coffee or tea (9) carbonated beverages (10) sweet juices (11) other……
10 Do you consume milk or any milk products? (1) yes (2) no
  Usually, your portion size consumed is? (1) 1 cup (2) 2 cups (3) more than 2 cups
11 Do you have a milk allergy? (1) yes (2) no
12 Are you vegetarian? (1) yes (2) no
13 Evaluate how many times do you consume the following:

- Milk (1 cup)
  (1) Never (2) less than once per month (3) 1–3 times per month (4) once per week (5) 2–4 per week (6) 5–6 per week (7) 1 or more per day
- Yogurt (1 cup)
  (1) Never (2) less than once per month (3) 1–3 times per month (4) once per week (5) 2–4 per week (6) 5–6 per week (7) 1 or more per day
- Cheese (slice)
- (1) Never (2) less than once per month (3) 1–3 times per month (4) once per week
- (5) 2–4 per week (6) 5–6 per week (7) 1 or more per day
- Sardine (3–5 ounce)
  (1) Never (2) less than once per month (3) 1–3 times per month (4) once per week (5) 2–4 per week (6) 5–6 per week (7) 1 or more per day

14 Evaluate how many times do you consume the following using frequencies (1–7) as used above?

**Table:**

# Frequency	weight	Portion size	Food item
	200 g	Cup	Broad bean
	20 g	10 pieces	Mint
	20 g	10 pieces	Parsley
	70 g	CUP	Red cabbage
	70 g	CUP	Cabbage
	150 g	1 piece	Tomato
	150 g	1 medium	Banana
	150 g	5 pieces	Apricot
	150 g	1 medium	Pomegranate
	30 g	1 ounce	Nigella sativa
	30 g	1 ounce	Sesame
	100 g	1 medium	Lemon
	100 g	2 large	Figs
	200 g	1 cup	Wheat
	30 g	1 ounce	Peanut
	200 g	cup	Soybean
	200 g	cup	Corn
	50 g	150 mL Cup	Dandelion
	150 g	1 medium	Potato
	100 g	1/2 cup	Cooked spinach
	30 g	150 mL Cup	Thyme
	50 g	3 pieces	Dates
	100 g	15 pieces	Grapes
	150 g	1 medium	Orange
	150 g	1 medium	Peach
	30 g	150 mL Cup	Horsetail
	60 g	Cup	Rocket
	150 g	5 pieces	Plums
	30 g	100 mL Cup	Coffee
	30 g	150 mL Cup	Green tea
