# Correction to “A Pilot Study Investigating the Influence of Dietary Boron Levels on Osteoporosis in Postmenopausal Women”

**DOI:** 10.1002/fsn3.71875

**Published:** 2026-05-11

Rababah, T., Aludatt, M., Gammoh, S., Salameh, F. B., Magableh, G., Almajwal, A., Yücel, S., AL‐Rayyan, Y., & AL‐Rayyan, N. (2024). A Pilot Study Investigating the Influence of Dietary Boron Levels on Osteoporosis in Postmenopausal Women. *Food Science & Nutrition*, 12, 5708–5721. https://doi.org/10.1002/fsn3.4218.

The first affiliation of the fifth author Numan AL‐Rayyan was removed from the article. The affiliation of the author should read as “National Agricultural Research Center, Amman, Jordan” in the article.

The online version of this article has been corrected accordingly.

We apologize for this error.

